# From “stuck” to satisfied: Aboriginal people’s experience of culturally safe care with interpreters in a Northern Territory hospital

**DOI:** 10.1186/s12913-021-06564-4

**Published:** 2021-06-04

**Authors:** Vicki Kerrigan, Stuart Yiwarr McGrath, Sandawana William Majoni, Michelle Walker, Mandy Ahmat, Bilawara Lee, Alan Cass, Marita Hefler, Anna P. Ralph

**Affiliations:** 1 Menzies School of Health Research, Charles Darwin University, PO Box 41096, Casuarina, Northern Territory 0811 Australia; 2 Royal Darwin Hospital, Darwin, Northern Territory 0811 Australia; 3 Flinders University, Northern Territory Medical Program, Darwin, Northern Australia 0815 Australia; 4 Aboriginal Interpreter Service, Northern Territory Government, GPO Box 4396, Darwin, Northern Territory 0801 Australia; 5 Charles Darwin University, PO Box 41096, Casuarina, NT 0811 Australia

**Keywords:** Cultural safety, Patient, Health, Aboriginal, Interpreters, Communication

## Abstract

**Background:**

Globally, interpreters are underused by health providers in hospitals, despite 40 years of evidence documenting benefits to both patients and providers. At Royal Darwin Hospital, in Australia’s Northern Territory, 60-90% of patients are Aboriginal, and 60% speak an Aboriginal language, but only approximately 17% access an interpreter. Recognising this system failure, the NT Aboriginal Interpreter Service and Royal Darwin Hospital piloted a new model with interpreters embedded in a renal team during medical ward rounds for 4 weeks in 2019.

**Methods:**

This research was embedded in a larger Participatory Action Research study examining cultural safety and communication at Royal Darwin Hospital. Six Aboriginal language speaking patients (five Yolŋu and one Tiwi), three non-Indigenous doctors and five Aboriginal interpreter staff were purposefully sampled. Data sources included participant interviews conducted in either the patient’s language or English, researcher field notes from shadowing doctors, doctors’ reflective journals, interpreter job logs and patient language lists. Inductive narrative analysis, guided by critical theory and Aboriginal knowledges, was conducted.

**Results:**

The hospital experience of Yolŋu and Tiwi participants was transformed through consistent access to interpreters who enabled patients to express their clinical and non-clinical needs. Aboriginal language-speaking patients experienced a transformation to culturally safe care. After initially reporting feeling “stuck” and disempowered when forced to communicate in English, participants reported feeling satisfied with their care and empowered by consistent access to the trusted interpreters, who shared their culture and worldviews. Interpreters also enabled providers to listen to concerns and priorities expressed by patients, which resulted in holistic care to address social determinants of health. This improved patient trajectories and reduced self-discharge rates.

**Conclusions:**

A culturally unsafe system which restricted people’s ability to receive equitable healthcare in their first language was overturned by embedding interpreters in a renal medical team. This research is the first to demonstrate the importance of consistent interpreter use for providing culturally safe care for Aboriginal patients in Australia.

## Introduction

Language is more than a communication tool; it is a pivotal aspect of culture which supports and strengthens Aboriginal and Torres Strait Islander people’s health and wellbeing [1–4]. Paradoxically, Aboriginal language speakers in Australia who have stronger social capital from speaking their first language often have restricted engagement with, and limited access to, English speaking health services resulting in poorer health outcomes [5]. Australia’s Northern Territory (NT) is one of the most linguistically diverse jurisdictions in the world [6]. Around 60% of the Aboriginal population speak one, or more, of dozens of Aboriginal languages as their first language [1, 6, 7]. The life expectancy of Aboriginal peoples in the NT is the lowest in Australia, around 15 years less than non-Indigenous people [8]. The prevalence of chronic diseases is also disproportionately high [9]. Research has found interpreters in health care improve patient outcomes [10], but in the NT interpreters are profoundly underused [11–13]. This is despite the formation of the NT Aboriginal Interpreter Service (AIS) in 2000 and literature spanning 40 years explaining the benefits of interpreters in healthcare. A 1979 federal government report stated “there is a desperate need” for interpreter services in NT hospitals, describing interpreters as a vital link in the communication chain between the “two nations” [14]. Unfortunately, since then culturally unsafe communication has continued contributing to growing suspicion and fear of health services, absence of informed consent and death [15–17]. Poor patient provider communication is also one of the most common ways patients experience racism [18–20]. Our previous research has found upwards of 50% of Aboriginal patients at Royal Darwin Hospital (RDH) would benefit from an interpreter, however currently an estimated 17% of patients get access [11]. Work to improve interpreter uptake at RDH, where most patients identify as Aboriginal, is underway however there remains considerable scope for improvement [11, 13].

This paper reports on a new model of Aboriginal interpreter use piloted at RDH in 2019. At the time of the pilot, RDH did not directly employ Aboriginal interpreters. Instead, depending on interpreter availability, the NT AIS provided one Aboriginal language interpreter to work at the hospital for 4 h every weekday morning. The ‘rostered hospital interpreter’ was not attached to a team or division but instead waited to be paged by a health provider [12]. These interpreters were underused and felt undervalued [12]. Additionally, Aboriginal interpreters could be booked with 24-48 h’ notice. However, because NT AIS interpreters worked across a range of settings including legal and community services they were often unavailable to work in the hospital when required. Recognising these problems, the NT AIS and RDH piloted a model in which Aboriginal interpreters were embedded in a medical team for 4 weeks. This guaranteed interpreters were consistently available face-to-face every weekday during the morning medical ward round in which critical medical decision-making moments occur. The model provided an opportunity to explore the impact of Aboriginal interpreters in the hospital on the delivery of culturally safe care. Proponents of cultural safety argue patient outcomes will improve when health systems no longer diminish and demean an individual’s cultural identity [21–27]. However in Australia, there is a dearth of evidence to demonstrate that culturally safe communication practices results in better patient outcomes [27, 28].

The aim of this paper is to present Aboriginal language speaking patient experiences and perspectives of hospital care when access to interpreter-mediated communication is consistent. The patient perspective provides a means to assess cultural safety, which by definition is determined by recipients of care [21]. Patient narratives are presented alongside stories from Aboriginal interpreters who share patients worldview and insights from non-Indigenous doctors regarding hegemonic thinking and systems. An in-depth examination of provider perspectives on the model will be presented separately (manuscript underway). The value of the approach presented here will inform the redesign of systems currently being explored by participating health and interpreter services.

## Methods

### Study design

This pilot study on Aboriginal interpreter-meditated communication at RDH is embedded in a larger Participatory Action Research (PAR) [29–31] project exploring the barriers and enablers to culturally safe communication at RDH [11, 12, 32, 33]. This PAR project entailed researchers and participants collaborating through a cycle of action and reflection to identify and address areas requiring transformation [29–31]. The theoretical framework was influenced by cultural safety [21] and critical race theory [34] which draws on Habermas’ approach to critical theory [35, 36]. The framework avoids problematizing Aboriginal peoples [37] and promotes “counterstories” [34, 38] which challenge and displace the narratives and beliefs that maintain inequities in colonised Australia.

### Researcher reflexivity

The lead author VK is an Australian born White researcher of Anglo-Celtic heritage; English is her first language. Reflecting on her White privilege [39–42] and capacity to assume what hooks refers to as the colonising role of the “privileged interpreter-cultural overseer” [43], VK uses PAR to facilitate the collective production of knowledge [29] to prioritise subjugated voices and challenge the status quo [34]. The second author SYM is a Gumatj man from the Yolŋu nation; Djambarrpuyŋu, a dialect of Yolŋu Matha, is his first language. SYM is an Aboriginal Health Practitioner and researcher. Through the moral bonds of kinship which are expressed as responsibilities and obligations [44] SYM was related to Yolŋu participants.

### Study setting

This pilot was a collaboration between Menzies School of Health Research, Top End Health Service (TEHS) and the NT AIS. TEHS manages RDH: a 360-bed tertiary referral hospital in the NT capital city, Darwin, on Larrakia country. The study was conducted in the RDH renal unit where approximately 90% of patients identify as Aboriginal. It was chosen due to the high percentage of Aboriginal patients and support from nephrologists. Due to unreliable language documentation at RDH [12, 45] the prevalence of Aboriginal languages spoken by renal patients was unknown. However, Djambarrpuyŋu, a dialect of Yolŋu Matha, is the most commonly spoken Aboriginal language in the region, with more than 4200 speakers from north-east Arnhem Land in the NT [1, 46]. For this reason, the NT AIS initially supplied a Yolŋu Matha interpreter to work alongside doctors. A Tiwi interpreter was also added after assessing the patient cohort. This supplemented the NT AIS on-demand interpreter service available at RDH as described above.

### Participant sampling

A purposeful sampling strategy was used to identify key informants who, as per PAR, had a vested interest in the area of study and could provide “information rich cases” which exemplified dysfunction and exposed opportunities for change [29, 30, 47, 48]. Also consistent with PAR, some participants were researchers. Aboriginal patients were eligible if they spoke an Aboriginal language as their first language, were hospitalised for a minimum 5-day period and able to consent to participate. Doctors, interpreters and interpreter support staff were sampled based on their commitment to study aims and availability (work roster).

### Data collection

Data sources for this study included interviews with patients, doctors and interpreters, researcher field notes, reflective journals by doctors, interpreter job logs and patient language lists. Semi-structured conversations with Yolŋu patients were conducted in Yolŋu Matha either by Yolŋu researcher SYM or by VK with an interpreter. Both SYM and the interpreters had perspectives on communication and health which aligned with those of patients [49] and relationships of accountability with patients through kinship [44]. These relationships ensured data was collected in a culturally safe manner [50]. Conversations were recorded at the hospital in the patients’ preferred locations, either at the beside or in a private room. Patients were thanked for their participation with a hospital café voucher. Doctors and NT AIS staff were interviewed before and after the pilot at a location of their choice by VK in English. Doctors’ journals and researcher field notes [47] documented participant experiences who consented to be observed only. Additionally, journals and notes were explored for contradictions and consistencies between patient, doctor and interpreter perspectives and used as a prompt for interviews.

With no reliable mechanism to document Aboriginal languages spoken by RDH patients [12, 45], participating doctors or NT AIS staff asked each patient: “what language do you speak at home?”. This was recorded on patient lists and shared with VK who entered de-identified data into an excel spreadsheet. To document the frequency of patient-interpreter-provider interaction, each interaction was logged by interpreters on the paper-based interpreter job log sheet. Handwritten data were double entered into an electronic database (Microsoft Excel v2011), by VK and a project officer then cross checked by VK for accuracy.

### Data analysis

Aligning with PAR’s transformative goals [51], a critical theory [36] lens guided analysis which was undertaken by SYM and VK. Interviews recorded in English were transcribed verbatim. Patient conversations recorded in Yolŋu Matha were interpreted into English by SYM. Throughout translation, SYM reviewed and shared contextual explanations, referred to as “cultural intuition” [52], which gave further insights into Yolŋu worldviews regarding health. Using NVIVO12, VK conducted inductive narrative analysis [53] of English transcripts to identify key turning points in patient trajectories of care. These turning points, refined through discussion with co-authors, were reconstructed into a consolidated patient journey which drew on patient, health provider, interpreter and researcher data. Consistent with critical race theory, this process revealed “counterstories” [34] which formed the basis of the findings. Pseudonyms were assigned for participants except for the specialist doctor (co-author SWM: Dr. William) and the NT AIS trainer (co-author MA: Mandy) who, as per PAR methods [30], were both researcher and the researched, and are both identified in accordance with their wishes.

### Ethical considerations

Pseudonyms derived from White first names have been used for the interpreters and patients who used their White names during interactions with health providers. We acknowledge the use of such pseudonyms risks perpetuating White cultural dominance however, following advice from Aboriginal researchers, Yolŋu or Tiwi pseudonyms have not been used as it risks compromising the cultural integrity of skin names which indicate a person’s bloodline and kinship obligations [54]. The term White is capitalised in line with Whiteness studies stemming from the sociological work of W.E.B Du Bois [55]. White does not refer to skin colour but instead refers to a social category which describes those who “participate in the racialized societal structure that positions them as “White“ and accordingly grants them the privileges associated with the dominant Australian culture.” [56] Regarding terminology, the nation or language group of Aboriginal participants will be used. Otherwise, the term Aboriginal, which refers to the original occupants of mainland Australia, will be used. Approval to conduct the study was provided by the Northern Territory Department of Health and Menzies School of Health Research Ethics Committee.

## Findings

The interpreter ward round pilot occurred in the RDH renal department in 2019 during two periods: 14th to 27th of August (10 days) and 25th November to 3rd December (7 days). Differences in duration was due to NT AIS resourcing issues. Seventeen interviews were conducted before and after the pilot. Shadowing of doctors by VK and SYM occurred between the hours of 8 am to 2 pm for a total of 29 h across 7 non-consecutive days, during which 20 patient interactions with interpreters were observed. In the RDH renal ward during the pilot 84% of patients identified as Aboriginal, of whom 78% spoke one or more Aboriginal languages. Fifteen Aboriginal languages, of which 13 were from the NT, were counted: the most spoken languages were Yolŋu Matha and Tiwi. Other languages documented were: Kunwinjku, Anindilyakwa, Kriol, Burarra, Murrinh-Patha and Ngan’gikurunggurr, Warlpiri, Maung, Wurlaki, Ngarinyin (Western Australia), Garawa, Yumplatok (Torres Strait Creole, Queensland) and Ngaringman.

Six patients participated. Yolŋu patients Patricia, Linda and Sally consented to be interviewed. Yolŋu man Paul and Tiwi man Owen consented to observations only. Yolŋu Elder Matthew, identified as a key informant, was interviewed three times and interpreter mediated healthcare interactions with Matthew were observed on 5 of the 7 days of shadowing doctors. SYM interviewed Matthew twice (2 months apart) during the 2019 pilot. As per PAR, a third conversation with Matthew occurred 18 months after the pilot to verify findings. The conversation, conducted by VK with a Yolŋu Matha interpreter, also allowed Matthew to add further details to his story. Yolŋu and Tiwi patient stories either support, or add to, Matthew’s perspectives and experiences. Additionally, three non-Indigenous doctors and five NT AIS employees all identifying as Aboriginal participated. Data were collected from two Yolŋu Matha interpreters, two Tiwi interpreters and an interpreter trainer. Doctor and NT AIS staff perspectives on the impact of the pilot are shared to locate the patient experience within the hegemonic institution. Consolidated patient journeys are presented below, which show key turning points in the patients experience due to regular access to interpreters.

We found consistent availability of face-to-face interpreters during medical ward rounds when critical patient care decisions were made positively shifted the patients’ experiences towards culturally safe care. Aboriginal language speaking patients who felt frustrated and misunderstood when forced to communicate in English reported feeling empowered and satisfied with the care they received with interpreters present.

### The frustrated and misunderstood patient

Without consistent interpreter mediated communication which allowed for clear two-way patient-provider communication, treatments were inflicted on frustrated, distressed and misunderstood patients. Some patients signed surgical consent forms without understanding what they were consenting to. We also found patients who experienced communication problems would self-discharge from hospital, exercising the limited power they had.

Yolŋu Elder Matthew had been a patient at RDH intermittently over 5 years. His home community was in north east Arnhem Land, 650 km away from the hospital. He had been in the English-speaking hospital system for so many years Matthew worried he was losing his language. Matthew was primarily under the care of the renal team but also being treated by other specialists for comorbidities. When health providers explained the reasons for his hospitalisation in English he said:

*“I was finding it hard when it was just me talking. Finding it really complicated and I would think to myself, ‘Oh who’s going to help me?’.” –* Matthew, Yolŋu Matha speaker.

Without an interpreter, Matthew wondered why doctors did not use plain English: “*I sometimes wonder to myself, are there simpler words they can use so maybe I can understand?”.* He explained he understood about 50% of what English speaking health providers say:

Matthew: *Half of it I don’t understand and then a little bit, I understand it a bit.*

SYM: *So when the interpreters came in, what did you think about that?*

Matthew: *It’s really nice…It really helps when Yolŋu are here and we both understand each other, and I understand. Sometimes I get confused and think ‘How am I going to talk?’ Really, I don’t know what to say.*

SYM: *Do you get angry alone?*

Matthew: *Yeah, I get angry when I don’t understand and sometimes, I think about praying and I pray to God about it because I’m so frustrated.*

Across the hospital, Matthew had a reputation as “*very abusive”* and “*very aggressive”*. It was argued *“that he understood English very well”* and he *“was being deliberately obstructive”* (Dr William, journal, 14/8/2019). Dr. Sean first met Matthew 4 years ago when he was “*very funny and witty”* but *“now he’s on dialysis, he’s pretty much bedbound. If he stands and walks, he’s in a lot of pain and he’s had recurrent infections.”* Over 5 years, Matthew underwent a series of major procedures, only partially understanding what was being done to him. Numerous surgeries were performed, including to enable his body to connect to a dialysis machine and to address comorbidities. One day before the pilot began, Matthew had surgery again. Dr. Sean worried whilst Matthew would nod in agreement when consent was requested in English, the display of gratuitous concurrence was just *“to get people to go away”.* Dr. Jack has seen other Aboriginal language speakers also undergo surgery without full comprehension of the procedure which was explained without an interpreter. He described it as *“unacceptable*”:

*“….and it’s not as if this is something benign. This is putting someone on a* (dialysis) *machine for the rest of their life and performing surgery to create access for that machine without explaining to them why.… and the same thing happens across the board. It happens in oncology. You give people…poison, toxic chemotherapy without really ascertaining as to whether they understand the risks and/or benefits.”* - Dr Jack

Interpreters also reported patients have attempted to withdraw written consent after the interpreter explained what the patient been signed:

*“when the doctor’s talking to them, they agree to everything and once I get there I ask them, ‘Do you know why you’re signing this form?’ And they go, ‘No’. That’s why it’s a bit difficult and I’m like, ‘You just signed a form for the doctors to take you to theatre’. And they’re like, ‘What? No, I don’t want to do that. Can you just rip the paper up?’.” –* Carly, Yolŋu Matha interpreter

Another example of how patients were misunderstood pertains to the label “frequent flyer”, which was given by staff to individuals who are frequently readmitted to hospital. Tiwi speaking patient Owen was one such patient. When health providers communicated with Owen in English, he would be discharged from hospital without the right supports in place to ensure he remained well. Owen was homeless and struggled with an alcohol addiction, but his circumstances were not well understood by the treating team. At times he would re-present to RDH so unwell he was admitted to the intensive care unit (ICU):

*“So he* [Owen] *would come in very short of breath and go into ICU. We’d start dialysis. He gets dialysis. He gets better. He’s discharged. He does the same. Until we did that ward round* [with interpreters]*” –* Dr William

Our findings also indicated that distressed misunderstood patients would self-discharge from hospital. Self-discharge results in prematurely stopping, or limiting, the medical treatment underway. Three reasons were identified as to why patients may self-discharge. Firstly, patients have responsibilities outside the hospital requiring attention. Linda had also been labelled a “frequent flyer”. With an interpreter present, she revealed that as the primary carer in her family, she needed to leave hospital to help her family address pressing legal and housing issues. Why Linda self-discharged had not previously been explored by doctors, which Dr. Jack believes is “*racist because we don’t explore why they want to walk out”.* Secondly, hospital priorities and procedures which shift across the organisation numerous times a day were not explained to the patient. Yolŋu patients Matthew and Linda were often frustrated after being told planned surgery was cancelled. Yolŋu Matha interpreter Carly said Linda was thinking about discharging herself because her surgery was repeatedly delayed:

*“She was saying that she was getting tired…*(they) *keep changing the times for her operation…she was ready to go home. But lucky I was there to explain to her all the stuff. Like, ‘There’s probably somebody in before you that’s got a worse condition than you’…She was like ‘Maybe that’s why they keep changing the times and not seeing me because there’s somebody else in front of me’.”* - Carly, Yolŋu Matha interpreter

Finally, frustrated misunderstood patients self-discharge because they felt uncomfortable or even persecuted. Dr. Jack said patients leave because the hospital is notoriously cold “*or it might be that they overheard someone saying that they smelled or they’re dirty*”.

None of the patients had requested an interpreter. Yolŋu patient Sally explained she had not requested an interpreter before despite a) having seen them on the wards at Gove hospital and b) stating she would prefer to communicate in Yolŋu Matha because doctors *“use too many big words and the interpreter helps me understand”.* Visibility of interpreters at RDH was low and many patients were unaware interpreters can assist at no cost to them. NT AIS trainer Mandy said the patient will not ask for an interpreter as *“they’re in a foreign place”,* potentially in pain, missing family and disempowered: “*They don’t understand that that’s their basic right to request the interpreter”.*

### Diminishing Aboriginal cultures and disempowering patients

In mid-2019 Matthew was admitted to RDH to treat a recurring leg infection. Six weeks into this 21-week admission, on Day 1 of the pilot, Matthew saw an interpreter for the first time. Matthew said before the pilot he did not have access to an interpreter in the hospital: *“there was nobody* (referring to interpreters) …*It was complicated, and I didn’t understand.”* He later added*: “I was very upset at that time”.* Yolŋu speaker Sally shared a similar story: she had been receiving treatment at RDH and Gove Hospital for over 2 years and said the first time she experienced interpreter-mediated communication in either Top End hospital was when interpreter Carly appeared with the renal team during the pilot.

Three reasons were identified for why Matthew had not been provided with an interpreter previously. Firstly, his Yolŋu surname which would have identified him as a Yolŋu speaker to interpreters was not on the hospital record. Matthew was registered at the hospital with his White and Yolŋu first names as his first and surname. On the first day of the pilot, Yolŋu interpreter Carly followed standard practice to identify Yolŋu Matha speakers: she studied the patient list for Yolŋu surnames. As languages were not methodically documented, interpreters assessed language needs based on surnames which link individuals to language groups. Carly identified two Yolŋu Matha speakers but Matthew’s incorrectly registered name meant he was not identified. Another patient was also missed this way. Matthew and the other patient were identified as Yolŋu Matha speakers in a second process of language identification undertaken during the pilot whereby Dr. Sean asked each patient directly what language(s) they spoke at home. Secondly, the ad hoc hospital system which relied on a health provider placing a magnetic removable ‘interpreter’ sign above the patient’s bed was not actioned for Matthew. During the pilot, if an ‘interpreter’ magnet was seen above a patient bed it was rare the name of the patient’s language was appropriately documented accompanying the sign. Five months after Matthew was admitted, and after his language needs had been clearly determined, there was still no language information above his bed, although there was a generic interpreter sign (VK field notes 26/11/19). Carly suggested if Aboriginal languages were visible on the ward, knowledge may improve:

*“I noticed that they don’t have, like, the language sign on top of their beds so maybe that’s why it was hard for doctors to find out where they came from and what language they speak.”* – Carly, Yolŋu Matha interpreter

The final reason as to why language discordance was not considered in relation to Matthew was because health providers assessed Matthew’s English as adequate.

*“At the start they didn’t get me an interpreter because they assessed my English and they said it was understandable. But when they got into the concepts of what had happened to my leg and what the treatment was, that’s when it got complicated and I didn’t fully understand”* – Matthew, Yolŋu Matha speaker

The same determination regarding English proficiency was made about Tiwi speaking patient Owen and Yolŋu Elder Patricia. Patricia said doctors assumed she was happy speaking English because she had previously worked as a lecturer at a Darwin college. Patricia explained, “*English is her second language*” and Yolŋu Matha is her “*normal language*”. During one bedside consult, Patricia declined an interpreter. From this single interaction, health providers extrapolated Patricia did not want an interpreter for any consult. The following day she explained to researcher SYM in Yolŋu Matha she was in pain the previous day and didn’t want to talk to anyone. She made it clear her preference was to speak in her first language with an interpreter present:

*“First language is important to us, it’s like growing up as a child into that language. That Yolŋu Matha it’s very important our language. We were born with it, we live with it, we prefer it.”* – Patricia, Yolŋu Matha speaker

Patricia suggested the reason why Aboriginal interpreters are not commonly accessed by hospital staff is because staff lack cultural competency. Both Matthew and Patricia said RDH staff require training to improve knowledge of Aboriginal cultures and the importance of speaking first languages: *“I want someone to deliver an awareness course here. In Yolŋu Rom.”* Researcher SYM explained that Patricia used Yolŋu in this case to refer to all Aboriginal peoples and Rom means “law” or “culture”. Patricia said Aboriginal and non-Aboriginal peoples should work together to deliver cultural education so both perspectives are understood.

### Building relationships of trust

When patients can communicate in their first language, they no longer feel frustrated or misunderstood. On Day 1 of the pilot, in the pre-ward round meeting, the renal team were warned about Matthew who had been tagged the “*angry man*” by hospital staff. He was the first patient on the list and so became the first to experience this new model of care. A large pack of providers (four doctors, the interpreter, the interpreter trainer, nurse in charge, two allied health team members and researcher VK) gathered around the “*angry man’s*” bed to see how he would react to the presence of an interpreter. Matthew was attached to the dialysis machine, the hospital-issued white blanket pulled up over his head. Patients commonly do this to get some privacy, block the fluorescent lights or to keep warm in the heavily air-conditioned hospital. With Carly interpreting, Dr. William introduced the assembled pack and told Matthew that today he can speak in his language. Matthew pulled the blanket down to reveal his face. What happened next was described by NT AISNT AIS trainer Mandy as “*mind blowing …as soon as he heard Carly’s voice in language, you know everything opened up”.*

During this initial interpreter-mediated bedside consult Matthew was able to comprehensively describe the pain he had been experiencing which resulted in doctors changing his medication. With an interpreter he also explained why he had a history of missing dialysis appointments at the hospital. Matthew said he had limited support at home and struggled to walk which meant he sometimes missed the hospital bus pick-up service to transport him for treatment. He told doctors he wanted to live in a supportive environment “*where I don’t get sick again.”* Dr. William said: “*We wouldn’t have picked that up without language.”* Although, the revelations were disputed by some staff who argued what Matthew said in English was more reliable than what was said in Yolŋu Matha through an interpreter. Dr. Sean journaled that disbelief may stem from some of his colleagues feeling like they had failed the patient:

*“Patients were able to tell us their true story for the first time after many months or years of work by professionals in the department, it is understandable that there is frustration that any work done by these professionals has been in vain.” –* Dr Sean, journal 15/8/19

The benefits of interpreter mediated communication for Yolŋu and Tiwi patients went beyond language interpretation. Reflecting on that first interaction with Yolŋu interpreter Carly, Matthew used the Yolŋu word *“latju”* (nice) to describe the experience. He said having another Yolŋu person present made him feel at ease: *“that was nice because Yolŋu were helping Yolŋu, really helping me.”* Matthew explained there is inherent trust between interpreters and patients who share a language because they are related:

*“The trust is massive…I feel when they come here* (interpreters), *I feel really good not only because I’m related to them, but I feel like the flow of the conversation is going faster. We are all understanding each other…. It’s just a good feeling when it’s flowing and everyone understands.”* - Matthew, Yolŋu Matha speaker

Dr. William said *“just having someone from the same community”* shifts the power imbalance between patient and provider. Doctors hoped that by working alongside trusted interpreters the patients who feared hospital would feel safer:

“*You know, getting Linda on-side. That we're not these terrible people and this is not the scary place where all her family members have gone to die.*” – Dr Sean

Yolŋu patient Sally said when she first meets an interpreter, she establishes her kinship relationship with them; this ensures both are clear on the responsibilities of their relationship which may include avoidance. Yolŋu Elder Patricia explained that because Yolŋu patients and interpreters share culture and beliefs, they can explain the unspoken subtext of the spoken words:

*“Like, this Balanda* [non-Aboriginal] *person doesn’t understand what this Yolŋu person is saying. So that’s why the Yolŋu has to be there to explain it to you. To make better communication with the Balanda people.”* - Patricia, Yolŋu Matha speaker

Yolŋu Matha interpreter Carly relayed an interaction, which did not occur during the pilot but during her prior experience as an interpreter, which showed how patient perspectives are understood by interpreters. During a consent discussion with a Yolŋu-speaking patient, Carly interpreted the risk of blood loss and the possibility of a blood transfusion. The patient was resistant to a blood transfusion because according to his ontology blood should not be transferred from one person to another:

*“Us Yolŋu people we don’t want to take other people’s blood and put it in our body, it’s just wrong because sometimes when you do that, like, you could have somebody’s family member going into you and what if that person had someone that was very close to him or her that passed away that was hanging around you and then you getting that in you, it would be hard.”* - Carly, Yolŋu Matha interpreter

Sharing the patient’s worldview, Carly was able to explain to the doctors the patient’s perspective. With this new information the treating team realised they needed to give the patient more time and information to consider all options.

### Proactive confident patients

Consistent access to interpreters meant Tiwi and Yolŋu patients were able to question the treatment offered, exercise choice and make decisions based on their priorities. Matthew’s power increased:

*“Yes I was more forceful with my treatment and making decisions and also I had more choices ….I was more forceful, making decisions based on things I wanted.”* - Matthew, Yolŋu Matha speaker

Two days after first experiencing the benefits of interpreter-mediated communication Matthew requested more information with an interpreter about his recurring infection. With Carly interpreting for Dr. Sean, a nuanced discussion regarding the complexities of infections, antibiotics and efficacy of antibiotic treatment occurred. Afterwards, Matthew said he was relieved because he finally understood his situation. He said: *“I could hear clearly.”*

As Matthew’s understanding of his health condition grew, he also became more confident communicating in English when interpreters were not available. On one occasion without an interpreter, he explored the option of moving to Sydney for treatment to be closer to his Sydney-based son, however *“the doctors said there is the same medication down there and here”.* Satisfied with the discussion and information provided, Matthew decided to remain in Darwin. Dr. William journaled (14/8/19) that with interpreters embedded in medical teams, patients became *“proactive partners”* instead of passive recipients of care unable to scrutinize the effectiveness of treatments*.*

Another example of increasing patient autonomy occurred in a family meeting. Yolŋu patient Paul had end stage kidney disease. He was faced with the life-or-death decision to start dialysis. Paul needed to speak with his family, most of whom were 580 km away in north east Arnhem Land. A video link was organised for Paul with his wife and children in Darwin to connect with family, who gathered at the remote community clinic. Unlike most family meetings where the clinical team controls the space, in this case Paul and his family were in control. Dr. Jack journaled on the 26/11/19: *“it was quite extraordinary. It was nothing like I’d seen before because we weren’t involved”.* The medical team faded into the background with the interpreter positioning herself behind Dr. William’s left shoulder. Interpreter Joanna was like an earpiece interpreter at the United Nations. For 25 min, one by one each person stood in front of the camera and spoke directly to Paul: they all encouraged him to try dialysis. Joanna whispered into Dr. Williams ear without interrupting the family’s conversation in Yolŋu Matha. Paul in his wheelchair listened to everyone, he said very little. After the meeting in which doctors encouraged Paul to try dialysis with a view to receiving a kidney transplant, Paul had 3 dialysis sessions and then decided not to continue. He wanted to go home to pass away. As Dr. Jack journaled (26/11/19) the benefit of interpreters “*wasn’t in explaining the medical details but the ability to listen to Paul’s concerns”*.

### Satisfied patients

Matthew received access to a Yolŋu Matha interpreter 11 times across 17 days. On some days Matthew saw an interpreter twice if attention was required from other members of the multidisciplinary team. Two months after first having access to an interpreter Matthew reported he felt much more supported: *“Yeah heaps of them are helping me”.* He added interpreters not only helped him understand; importantly they helped the health providers understand his perspective and priorities. Matthew said with interpreters, communication works both ways: *“None of us are stuck or confused.”* Being able to communicate in his first language, Matthew was able to express his needs beyond the acute conditions he was being treated for. This resulted in addressing the social determinants Matthew articulated in the initial interpreter mediated consult. He received occupational therapy to improve movement, housing assistance and also a change to his hospital diet. Considering Matthew was hospitalized for nearly 5 months, food was a significant part of his hospital experience: *“I wasn’t eating the hospital food. I would just buy food from the* (hospital) *cafe.”* Matthew was a saltwater man, from a remote island community where fish was an important part of his diet. With an interpreter present he requested fish once a week via the hospital dietician. Carly shared her conversation with Matthew:

*“(*He said) *‘I just want to be home and have fish and something I feel comfortable eating’….and then he asked me like, ‘Why hasn’t this happened before? I was here for how long? And nobody spoke to me about my food but I’m happy that you came’.”*– Carly, Yolŋu Matha interpreter

Interpreter Joanna explained for Matthew, who is hundreds of kilometres away from his family, just being able to communicate in his first language “*cheers him up instead of trying to speak English”.* Staff attitudes towards Matthew changed. Dr. Sean said: “*once Matthew knew what was happening to his body, he suddenly was no longer this ‘angry man’ that everyone talked about”.* Dr. William reported Matthew’s trajectory changed:

*“Now Matthew he’s done very, very well. He’s been discharged. He’s living in some accommodation of his choice because that was one of the things which we didn’t understand but he explained it through the interpreter. He completed his courses of antibiotics which he would have missed some if he hadn’t, if things hadn't been explained to him. He didn’t understand why he was taking the antibiotics. So that was a huge change in his life.”* – Dr William

During the pilot, doctors noticed patients who were previously referred to as “frequent flyers” were now attending dialysis regularly and therefore not being readmitted through the emergency department. After the purpose of dialysis was explained to Tiwi speaker Owen, and he was also able to explain his personal circumstances, he was discharged into an alcohol rehabilitation program and began attending dialysis regularly. After Yolŋu patient Linda, who was stuck in a pattern of self-discharge and readmission, voiced the legal and housing issues her family faced, the treating team arranged for support staff from relevant external services to attend the hospital with an interpreter to solve the problems. Linda said she valued having an interpreter to assist in solving the non-clinical issues which were affecting her ability to engage with clinical care. Linda stayed in hospital for 9 days after her priorities were addressed. Participating doctors asserted a simple cost analyses would show regular interpreter use will save money on admissions:

*“So for Owen and Linda, if spending $100 on an interpreter every day even prevents one $20,000 ICU admission, I think it's worth it.”* - Dr Sean

At the end of the pilot, doctors reported Yolŋu and Tiwi patient projected health outcomes improved. Dr. Sean said: “*we’ve completely changed trajectories of illness and probably will save lives based on this project.”* All patients preferred to speak their first language in the hospital. Matthew said before interpreters became involved in his care he was *“stuck”* but after consistent interpreter-mediated communication with staff he is *“satisfied”.* Yolŋu Elder Patricia wants to see the model of embedded interpreters in medical teams permanently: “*Balanda doctor and Yolŋu interpreter all the time. I need to see that happen.”*

## Discussion

To our knowledge, this is the first study to qualitatively document the Aboriginal language speaking patient experience of culturally safe care in an Australian hospital. Cultural safety advocates for changing systems, and attitudes, which enables a transfer of power from service provider to health care consumer [21]. We found Tiwi and Yolŋu hospitalised patients who were frustrated and misunderstood became empowered after receiving consistent access to interpreters. Aboriginal interpreters who shared patient worldviews acted as cultural brokers, bridging the gap between western medicine and Indigenous knowledges [3, 13, 14] as well as provided linguistic interpretation. Changing hospital systems to ensure access to Aboriginal language interpreters, albeit for only 4 weeks, also changed patient health trajectories. Our findings contribute to research which asserts Aboriginal patients want Aboriginal providers involved in their care [57] and that language is a vital expression of cultural identity with demonstrated benefits for health outcomes [1, 2, 4, 5].

Yolŋu and Tiwi patients were gladdened by the presence of Aboriginal interpreters who were seen as a trusted ally. Interactions with health services for Aboriginal peoples are shaped by experiences of racism and powerlessness [58]. When engaging with mainstream services, many Aboriginal peoples anticipate racism regardless of whether they have been discriminated against [59]. We found Aboriginal interpreters, related through kinship to patients, provided a shortcut to developing trust with the patient. Aboriginal language interpreters came to work at RDH with a bank of social capital [60] stemming from kinship relationships which have high standards of “responsibility, with special attention to relationships of care, reciprocity, and consent, among others.” [44] As Tiwi and Yolŋu patients had trusting relationships with interpreters, the impersonal nature of the large ward round with rapid fire clinically focused questions [61] changed. The usually intimidating interaction with a large medical pack standing over a patient’s bed, shifted to a style more akin to “the reciprocal nature of yarning” [37], in which patients developed a comprehensive understanding of care which enabled them to communicate in English when an interpreter was not available*.* Patients were strengthened by kinship, shared knowledge and adherence to cultural protocols: this is a culturally safe service.

When patients speak their own language, they can exert control in an environment where they may otherwise feel disempowered. With interpreters present, patients were able to verbalise their priorities which included food. Food provides emotional as well as nutritional sustenance [21] and unappetising food adds to the discomfort of the hospital experience [12]. For other patients, expressing priorities meant social determinants affecting their capacity to engage with healthcare were addressed. This led to a reported drop in readmission rates and self-discharge. In the hospital, self-discharge is often the only form of resistance a patient can utilise against a culturally unsafe service, therefore self-discharge rates can be used as an indirect measure of cultural safety [27].

Consistent interpreter mediated communication meant that Yolŋu and Tiwi patients developed a more comprehensive understanding of their condition and hospital processes. In primary health care, the use of English with Aboriginal language speaking patients has been found to be inadequate as it failed to communicate “often lifesaving information to clients” [62]. Our research found the same evidenced by patients who, before the pilot, had a history of repeated admissions to the ICU. Unable to communicate in their first language, patients were disengaged from their care, and medical outcomes were suboptimal [13], and lives were at risk. However, with interpreters working alongside doctors over several days, patients were better able to consider the information delivered and question their treatment. Patients also felt empowered after hospital processes were clearly explained by a trusted source. When a patient’s surgery is repeatedly cancelled, that can be interpreted as disrespect and even discrimination. Understanding their clinical condition, and hospital systems, empowered patients to lead decision-making, including going against medical advice in favour of spending time on country with family before passing away.

Culturally respectful communication is a key component of delivering culturally safe care [21, 26, 63]. Before interpreters were embedded into the renal team, provider communication with Aboriginal language speaking patients could be described as ranging from pragmatic to hostile, as indicated by patients being labelled “*angry*”. When miscommunication occurred, the patient was blamed and consequently labelled non-compliant or non-communicative. Similarly, the labelling of patients as “frequent flyers” triggered another negative stereotype that Aboriginal patients were not interested in maintaining their own health [26]. These labels assisted in socialising other staff into expecting Aboriginal patients to be difficult: such attitudes support individual and institutional racism. We found once health providers took responsibility for communication by changing systems, the perception of the non-compliant angry patient was overturned and the so-called “frequent flyers” stop re-presenting to hospital. Our research shows that when health providers invest time listening to and communicate with patients, rather than speaking about patients, health outcomes improve. Further research into the perception that time spent with patients is costly is required to ensure health providers are not engaging in “false economies” [26].

Whilst English is the operational language of TEHS, it is not the language most spoken amongst renal patients. Almost 90% of patients were Aboriginal and nearly 80% spoke one or more of the 15 languages identified in the unit. A culturally safe service is actively mindful and respectful towards Indigenous cultures, strengths and differences [27]. To that end, we recommend the following changes to hospital processes and systems to ensure cultural determinants of health are addressed. Firstly, health providers make incorrect assessments about the need for an interpreter, a finding supported by previous work in this setting [64]. The NT AIS asserts that the main purpose of the interpreter is to allow the health providers to speak the patient’s language. This approach removes the need to judge the patient’s English proficiency because it is the language proficiency of the health providers which should be judged. If the health provider does not speak the patient’s language fluently, an interpreter is recommended. This is culturally safe, person-centred care. Secondly, language documentation must be addressed immediately. Previous research found that language was documented for only 44% of Aboriginal patients and in some cases, languages were identified as “Aboriginal” or “local” language reflecting the lack of importance staff place on information [12]. Additionally, there were seven separate RDH administrative and clinical forms which provided space to document patient language [12]. Of those seven forms, one of the most used forms, the patient list was not included. The patient list was used by doctors and the multidisciplinary team from the start of their shift, and consistently throughout the day in the process of care delivery. We recommend language be documented on the patient list alongside name and date of birth. This would ensure language discordance is considered at the same time as clinical discussions and it would also improve familiarity of Aboriginal languages in the NT. Thirdly, poor language documentation may be due to the low level of awareness of Aboriginal languages in the NT. Both cultural competency and cultural safety training should be regularly undertaken to improve awareness of local Aboriginal cultures, cater for the high turnover of staff and to show that the institution values culturally safe communication [33]. Clinical competencies, technical expertise and theoretical knowledge prioritised by institutions are only part of delivering comprehensive care [65]. Finally, we recommend that patients should be registered with healthcare facilities using their correct names, not their colonised names. Names give people an inalienable connection to country and kin [59] hence interpreters can assess language needs based on a patient’s surname. The format of Australian legal documents often forces name changes to conform with White norms which is a form of assimilation [59].

### Limitations

Findings may under-represent the prevalence and diversity of Aboriginal language speakers during the pilot for two reasons: patient language details were undocumented on one day of the study and Yolŋu Matha and Kriol were each counted as single languages during data collection. Yolŋu Matha and Kriol are umbrella terms for a collection of mutually comprehensible dialects and languages. We also recognise that reporting on a small sample size does not technically permit broad generalizations. However, logical generalizations can be made from the evidence produced [40] which is representational of other Aboriginal language speaking patients in the same setting. Hospitals can improve the quality of care by exploring and understanding the patient’s insider perspective revealed through key informants [66].

## Conclusion

The United Nations Declaration on the Rights of Indigenous Peoples, adopted by the Australian government in 2009, has enshrined the right for individuals to “understand and be understood” in their first language and if not, the state must ensure “the provision of interpretation” [67]. It is clear from our findings the state has failed to provide services to Aboriginal language speakers requiring hospital care in the Top End of the NT. Changing systems to facilitate easy access to Aboriginal language interpreters in the hospital addressed an institutionally racist system [68]. Implementation of a model of care comprising Aboriginal interpreters embedded in medical ward rounds achieved transformative change in patient experience. Patients described that the frustrations of hospitalisation, characterised by misunderstandings and distress regarding their diagnoses, treatment options and hospital systems, were overcome when an interpreter was included in the multidisciplinary team. Clear communication in first language averted premature discharges and allowed patients to make decisions according to their priorities. An enabling health system which places interpreters at the coal face of care delivery was shown to be essential for the provision of culturally safe care. Health care delivered in the absence of this approach – as experienced by the participating patients before the study - was unsafe and ineffective.

## Data Availability

Data collected and analysed during the current study are not publicly available due privacy issues and ethical considerations. Data may be available from the corresponding author on reasonable request.

## Abbreviations

ICE: Intensive Care Unit
NT: Northern Territory
NT AIS: Northern Territory Aboriginal Interpreter Service
PAR: Participatory Action Research
RDH: Royal Darwin Hospital
TEHS: Top End Health Service
